# Improved Discovery of Molecular Interactions in Genome-Scale Data with Adaptive Model-Based Normalization

**DOI:** 10.1371/journal.pone.0053930

**Published:** 2013-01-22

**Authors:** Julia Salzman, Daniel M. Klass, Patrick O. Brown

**Affiliations:** 1 Department of Biochemistry, Stanford University School of Medicine, Stanford, California, United States of America; 2 Howard Hughes Medical Institute, Stanford University School of Medicine, Stanford, California, United States of America; 3 Department of Statistics, Stanford University, Stanford, California, United States of America; The John Curtin School of Medical Research, Australia

## Abstract

**Background:**

High throughput molecular-interaction studies using immunoprecipitations (IP) or affinity purifications are powerful and widely used in biology research. One of many important applications of this method is to identify the set of RNAs that interact with a particular RNA-binding protein (RBP). Here, the unique statistical challenge presented is to delineate a specific set of RNAs that are enriched in one sample relative to another, typically a specific IP compared to a non-specific control to model background. The choice of normalization procedure critically impacts the number of RNAs that will be identified as interacting with an RBP at a given significance threshold – yet existing normalization methods make assumptions that are often fundamentally inaccurate when applied to IP enrichment data.

**Methods:**

In this paper, we present a new normalization methodology that is specifically designed for identifying enriched RNA or DNA sequences in an IP. The normalization (called adaptive or AD normalization) uses a basic model of the IP experiment and is not a variant of mean, quantile, or other methodology previously proposed. The approach is evaluated statistically and tested with simulated and empirical data.

**Results and Conclusions:**

The adaptive (AD) normalization method results in a greatly increased range in the number of enriched RNAs identified, fewer false positives, and overall better concordance with independent biological evidence, for the RBPs we analyzed, compared to median normalization. The approach is also applicable to the study of pairwise RNA, DNA and protein interactions such as the analysis of transcription factors via chromatin immunoprecipitation (ChIP) or any other experiments where samples from two conditions, one of which contains an enriched subset of the other, are studied.

## Introduction

Identifying the sequences specifically bound by RNA and DNA binding proteins is an area of intense investigation. The importance of transcription factors and other DNA binding proteins in regulating RNA expression has been appreciated for decades. After transcription, mRNAs interact with RNA-binding proteins (RBPs) that control their processing, localization, translation, and eventual decay. There is growing evidence for the existence of an extensive post-transcriptional regulatory network, mediated in part by RBPs [1], [2], [3], [4], [5]. It is likely to be the combinatorial, coordinated, and programmed interactions with specific sets of RBPs that are responsible for the gene-specific life history of each RNA after it has been transcribed [5].

Statistically sound methods for identifying specific RNA targets of RBPs are thus important to understanding biological regulation. There is a close analogy between this statistical problem and problems arising in studies of differential gene expression, with one major and fundamental difference being the treatment of normalization. Despite a large literature on the normalization of microarray data [6], [7], [8], [9], including situations where a majority of genes are expected to be differentially expressed [10], [11], little attention has been paid to normalization for experiments where *a priori* anywhere from a handful to the majority of genes could be differentially enriched in the IP compared to the experimental control (which we will call the “Mock”).

By imposing a scientifically appropriate assumption of “enrichment” of one sample distribution over another, we are able to make progress in modeling that leads to a novel normalization procedure. The method we propose is an intuitive method of normalization for microarray or high throughput sequencing experiments, which can be applied to situations where a minority or a majority of genes are differentially enriched.

Recent biological insights have been gained by characterizing the species of RNA bound by particular RNA binding proteins (RBPs) via microarray studies. Briefly, an RBP of interest is purified by immunoprecipitation (IP) with a specific antibody directed against the RBP or by affinity purification via a fusion of the RBP and an affinity tag. The RNA that co-purifies with the RBP is compared by microarray hybridization or high-throughput sequencing to the non-specifically bound RNA measured from the same IP procedure done without the specific antibody or using lysates from an untagged strain (called the Mock). The use of microarrays for identifying the targets of an RBP (see [1], [2], [3], [4], [12], [13]) generally proceeds as follows: hybridization to the microarray is performed to compare the RNA resulting from the IP of the RBP of interest (or a Mock IP) labeled (for example) with Cy5 (red), against a measurement of the expression of each mRNA in a total RNA sample from the cell lysate labeled (for example) with Cy3 (green). The ratio of Cy5 to Cy3 fluorescence (in this example) represents a measure of the enrichment of each RNA in association with a given RBP, and the corresponding ratio in the Mock IP experiment represents a measure of the RNA non-specifically enriched by the same procedure in the absence of a specific antibody or a tagged protein. These experiments are often performed in replicate. Since RNA may bind non-specifically during the IP procedure, the statistical challenge is to use the measure of the background (the Mock) along with the IP to identify RNAs that interact specifically with the RBP. Numerous studies [1], [4] have relied on this experimental design, using a standard statistical methodology for identifying targets, which is outlined below.

The normalization used when comparing two samples is especially important for IP enrichment experiments because a critical question is which distinct set of RNAs interacts specifically with a given RBP, and are thus differentially enriched between the IP and the Mock. One simple way to identify genes with differential enrichment factors between the Mock and IP is the t-test. The t-test is inherently sensitive to the normalization of results for the two samples being compared. The choice of normalization procedure therefore critically impacts the number of RNAs that will be identified as interacting with an RBP at a given significance threshold. The main contribution of this paper is to present a novel normalization procedure, which we argue is less biased and makes fewer and more justified assumptions than previous methods.

In the past [1], [4], the statistical approach used to identify the enrichment of sequences in the IP compared to the Mock was identical to the approach used if the two groups of data were derived from a study of differential gene expression: by making the assumption that the mean or median (or some other quantile) of the fluorescence ratios measured by microarray hybridization was equal between the Mock and IP experiments (a convenient assumption but a debatable one, even for global gene expression data). There is scientific reason to believe that this assumption is fundamentally inaccurate: the fluorescence ratios measured for RNA isolated by an RBP affinity selection should be a superposition of those signals in the Mock (i.e. due to spurious factors in the procedure) with some signal (if any) due to an interaction between the RBP under study and a subset of RNAs. Other normalization procedures (e.g. [6], [9], [14], [15]) are also inappropriate for the model that an IP is a superposition of a Mock and true signal.

We present a novel normalization methodology that takes into account an explicit and, we believe, generally scientifically justified, model of the IP – Mock experimental design. We find that this method gives a biologically coherent characterization of targets of several RBPs, which is sometimes quite different from that obtained using median normalization procedures. For example, [4] reported the targets of 42 RNA binding proteins in yeast, including poly(A) binding protein (PAB1). PAB1 is a highly abundant RBP that recognizes a poly(A) sequence found on nearly all mRNAs, it is essential for the translation of mRNAs, and consequently it has been suggested, with good evidence, that PAB1 may bind to nearly all polyadenylated mRNAs in the cytoplasm [4], [16].

Among the more than 4,000 transcribed yeast mRNAs detected in the experiment considered in [4], only ∼1,300 were identified (using a false-discovery-rate (FDR) threshold of 1%) as being targets of PAB1 using median centered normalization followed by Significance Analysis of Microarrays (SAM) [17], although the same authors provide independent, strong evidence that PAB1 may bind to nearly all mRNAs. In contrast, the AD normalization method (as outlined in the Methods section) followed by SAM finds more than 3,500 targets of PAB1–much more consistent with the independent, strong evidence that PAB1 may bind to nearly all polyadenylated mRNA in the cytoplasm. (see Table 1).

**Table 1:** Number of targets called by SAM After AD or Median normalization.

RBP	Median Normalization	AD Normalization	% of Detectable mRNAs
PUF1	33	27	0.45%
PUF2	377	301	5.0%
PUF3	303	338	5.6%
PUF4	319	306	5.1%
PUF5	413	305	5.1%
PAB1	1371	3511	81%

The AD normalization method enables the identification of more targets for RBPs with many targets, and a comparable number of targets for RBPs with few targets. The number of targets identified by SAM after the data were normalized by either median normalization or AD normalization is shown for the RBPs PUF1, PUF2, PUF3, PUF4, PUF5, PUB1, and PAB1. Also shown is the percent of the total number of detectable mRNAs that were called as targets after AD normalization.

Not all RBPs have a higher number of targets when AD normalization is used, as compared to median normalization. For example, some RBPs with small, distinct target sets, like PUF3, have a similar number of targets identified as significantly enriched, regardless of whether AD or median normalization is used (Table 1).

## Methods

### Methodology Overview

The normalization methodology presented here is a statistical method ideally suited for experiments where samples from two conditions, one of which contains an enriched subset of the other, are studied with the aim of identifying the enriched subset. For our purposes, an RNA species is considered “enriched” if its abundance is increased relative to a total RNA sample following an IP of an RBP of interest, suggesting it is specifically bound by the RNA binding protein being purified.

The AD normalization procedure is described here in plain language, with a formal mathematical description following. 1) For each entity being evaluated (e.g. an RNA or a DNA fragment), we calculate the average median centered enrichment value in the Mock experiment. 2) We identify the set of genes with the greatest difference *in the average Mock relative to a given IP replicate* (i.e. the greatest Mock – IP values). The size of this set of genes is chosen in a disciplined way described below. This is the set of genes that are the least enriched in the IP relative to the Mock, suggesting that they are non-targets whose enrichment is due primarily to non-specific binding. 3) We then normalize the given IP replicate so that the median of this set of non-target genes is the same as it is in the average Mock – based on the assumption that the non-specific enrichment of these non-target genes remains constant between Mock and IP experiments. 4) The same procedure is applied to the Mocks as a control, by removing one Mock replicate from the full set of Mocks and normalizing it relative to the other remaining Mocks as if it were an IP replicate (i.e. the well-known statistical “leave one out average”). We use this control for the Mock to estimate the statistical bias of our method when applied to the IP data.

The methodology is designed for experiments using an explicit model of background binding which includes an empirical measurement of that background. The model is based on the intuitive observation that an RNA's enrichment as measured by microarray hybridization or deep-sequencing of an IP-selected RNA sample, can be represented in terms of the enrichment due to background binding as estimated by the Mock (or averages of such arrays) superimposed with a signal that represents enrichment due to the IP and modeled using the simple statistical framework:
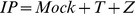
(1)where *IP* is the enrichment of the RNA by the specific IP, *Mock* is the apparent (spurious) enrichment by the Mock procedure, *T* is non-negative true signal (the variable of interest), and *Z* is a random mean zero error representing stochastic noise in the experimentally observed signals. In practice, these quantities are estimated with one or more experimental replicates.

Estimating T is non-trivial because the *Mock* and *IP* are only known up to scalar shifts (addition) of normalizing constants that themselves cannot be observed. Modeling these normalizing constants (*c_IP_* and *c_Mock_* for the IP and Mock respectively) produces the following restatement of Equation (EQ1) in terms of observable signals and unobserved normalizing constants:
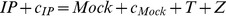
(2)


The statistical problem is to estimate *c_IP_* and *c_Mock_*. Equation (EQ2) demonstrates that accurate estimation of the difference between *c_IP_* and *c_Mock_* are essential and sufficient for making subsequent analyses that identify IP over Mock targets relatively straightforward. For example, after estimating *c_IP_* and *c_Mock_*, the corrected values could be subjected to a modified t-test procedure such as SAM with two groups: *IP_i_ + c_IP_ − (Mock + c_Mock_ )* for each IP experiment and *Mock_j_ + c_Mock_* for each Mock experiment [17]. This formulation shows that for the purpose of the t-test (and its modifications by SAM), it is sufficient to estimate *c_IP_ − c_Mock_* and to assume that the median of *Mock* is zero.

### Adaptive Approach

The main contribution of our method is the procedure to produce an alternative to a mean or quantile based estimation of *c_IP_ − c_Mock_*. A reason to avoid mean or median normalization is that doing so implicitly assumes that some values of the expectation of *T* are negative: after median centering, some of the Mock will necessarily be enriched with respect to the IP, countering the assumptions of the IP enrichment experiment. The basic idea of AD normalization is as follows:

Observe that after normalization, the only enrichment in the Mock compared to the IP should be due to noise, i.e. if the c's are correctly estimated,
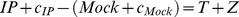
and *T* is a non-negative.

**Figure 1:**
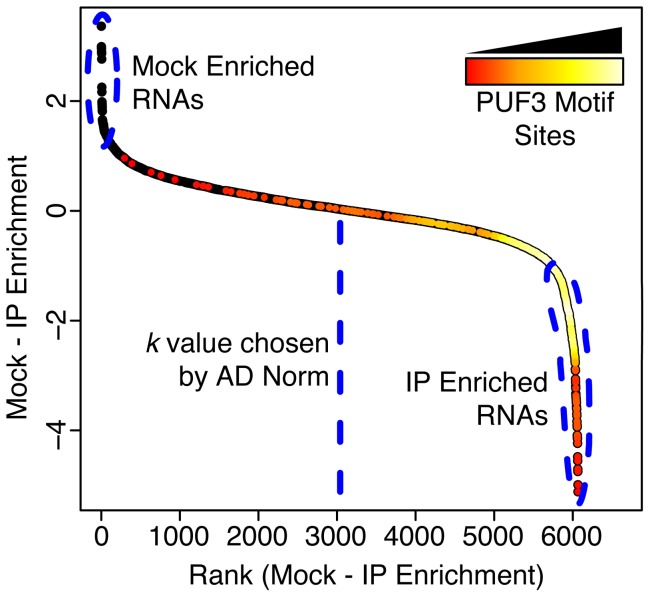
An example of the plot used to find *k.* An example (for PUF3) of the plot used by the AD Normalization method to find the number of genes to use for normalization (*k*). Each spot is an individual RNA. The y-axis shows the Mock-IP enrichment values and the x-axis shows the rank of those same values. All mRNAs are plotted with black circles. The heat colors show the density of mRNAs that contain a PUF3 motif site in their 3′-UTR (no motif: black, with motif: from orange to yellow). The vertical section on the left (indicated with a blue dashed circle) corresponds to RNAs that are most enriched in the Mock relative to the IP. The vertical section on the right (indicated with a blue dashed circle) corresponds to RNAs that are most enriched in the IP relative to the Mock. The vertical dashed line indicates the *k* value chosen by the algorithm. All the RNAs to the left of this line were used to normalize this array. 91% (311/343) of the PUF3 3′-UTR motif site containing mRNAs fall to the right of this line, suggesting that our algorithm was successful in identifying the primarily non-target (i.e. background) RNAs to use for normalization in this case.

Furthermore, the mean of *Z* is assumed to be zero. On this basis, RNA species that are the most relatively enriched in the Mock compared to the IP are most likely to be true background, i.e. have true signal equal to zero. These RNAs are identified by ranking the component-wise differences of the vectors (*Mock – IP*) and selecting the *k* RNAs with highest rank (Figure 1). The parameter *k* is the number of RNA species, DNA fragments, etc. that are assumed to be non-targets and therefore used for normalization (the process of choosing *k* is discussed later in detail). In practice, we have identified these RNAs by ranking the differences 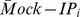 where 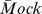 is the average enrichment among the Mock experiments and 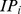 is an individual IP replicate. Call the set of RNAs identified in this way *S_k_*. Once these RNAs are identified, the Mock and IP can be normalized to the mean or median of *S_k_* in each condition, i.e.
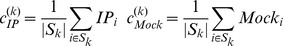



The above procedure introduces two obvious biases that we address in the implementation of the AD method:

1. Because the choice of *k* affects the value of each normalizing constants 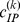 and 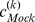, a disciplined method for identifying *k* is presented.
2. If two technical replicates were subject to the normalization procedure, one being normalized with respect to the other, (for example if an IP experiment were replaced by a Mock), by the nature of the naïve estimation procedure above, we would expect the computed 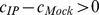.

We address 1) and 2) by modifying the naïve estimation of:
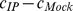
in such a way that the estimate is asymptotically unbiased, and for finite samples results in a conservative identification of IP enrichment relative to the Mock.

### Normalization Procedure

The formal description of the normalization procedure that was described above is as follows:

1. Compute the average of the median centered mock arrays 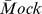, and the leave-one-out average mock, for each *Mock, 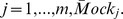
*
2. For the *j^th^* IP experiment define 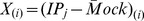, for 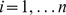, i.e. the order statistics of 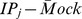. Compute the normalization constant for the *j^th^* IP:

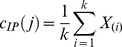

3. Define the *j^th^* normalized IP experiment 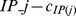.
4. For each Mock experiment, 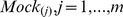, define 
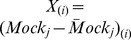
, i.e. the order statistics of 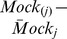.Compute:

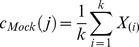

5. Define the *j^th^* normalized Mock experiment as 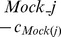.

After the last step, the data can be input into any statistical procedure for identifying enriched genes in the IP compared to the Mock. For example, we ran two class SAM on these normalized data and compared the results to those of SAM run on median centered data in the next section.

The following 3 important statistical properties of the normalization method are stated and proved below. In summary, Property 1 shows that our normalization method is not affected if all data points from any replicate are arbitrarily shifted by a constant, as may occur in microarray data from technical scanning effects: our procedure will always give the same result if array values are multiplied by an arbitrary constant and then log transformed. Property 2 shows that as data quantity increases, our estimator becomes more and more accurate.

Property 3 shows that the method does not overestimate the difference in normalizing constants between the IP and mock – doing so would cause more targets to be called significant than actually are significant.

1. The normalization constant is invariant to addition of a constant to each RNA's measurement value and invariant to addition of a common constant to each Mock array. For the first part, it is clear that if a constant c is added to each RNA's value, then the calculated normalization constant in the AD method will be increased by c, and the resulting normalized IP values will not be changed. Since the Mock arrays are median centered, addition of a common constant to the Mock will not affect the average Mock value.
2. If all RNA sequences measured in the IP are actually background, the estimate of the difference between the normalization constant for the IP and any Mock normalization constant is asymptotically unbiased. To see this, denote the differences between RNA values in the IP and Mock as random variables 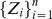 and the differences between the Mock and the leave-one-out Mock are 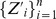. Then the AD estimate of the normalization constant from one IP replicate with k RNAs is equal to
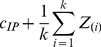
(3)where 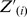 is the estimate of the *i^th^* order statistic of the difference between the IP and the average Mock. Similarly, the AD estimate of the normalization constant from one Mock replicate with k RNAs is equal to
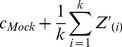
(4)where 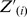 is the estimate of the *i^th^* order statistic of the difference between the *j^th^* Mock and the average Mock leaving j out. Since under the null hypothesis that no RNA is enriched by the IP, 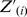 converges a.s. (almost sure, a measure of convergence in probability) to 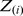 as the sample size goes to infinity, the continuous mapping theorem shows that 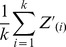 converges to 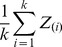 a.s. and hence the difference between Equations (EQ3) and (EQ4) converge to *c_IP_* – *c_Mock_* [18].
3. If not all RNA values in the IP are actually background, the estimate of the normalization constant is asymptotically biased in such a way that a t-test using normalized data is conservative. If not all RNA values in the IP are background, then it is easy to see that a.s.,

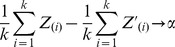
where *α* is a constant less than zero. Intuitively, *α* <0 if not all RNA values are background, because the distribution of differences between the Mock and IP will be more negative than when the IP is actually all background. This implies that the order statistics of the Mock-IP will also be more negative than when the IP is actually background. So, their expectation of the order statistics satisfies the following inequality:



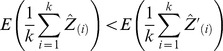



Underestimating *c_IP_ − c_Mock_* in a t test will result in a conservative estimate of significance.

### Choice of the number of genes to use for normalization (*k*)

The statistical procedure and analysis outlined here for estimating the normalizing constant is valid for any choice of *k*. For consistency of our analysis between different IPs, we followed a disciplined and unsupervised procedure for choosing *k* based on an empirical linear regression model as described below.

For the diverse data analyzed in this paper, the distribution of order statistics of the differences of Mock-IP RNA values followed a distribution which can be approximated as a piecewise linear function with each piece having a goodness of fit of 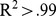 and knots separated by at least 500 x-axis values. In particular, this function was approximately linear between the 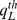 and the 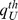 quantile for small values of the 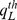 quantile of the distribution and large values of the 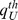 quantile (Figure 1). This method was empirically successful for all of our data and all of the publically available data we analyzed.

Under the assumption that the lower (left hand) tail behavior of the Mock-IP distribution is governed by the tail behavior of the order statistics of true background genes, it will not be piecewise linear in the x-axis until the 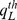 quantile of the background genes has been reached as we have empirically observed that the Mock-IP distribution is not linear in that region.

By finding the point on the x-axis in the plot of the IP-Mock order statistics where the plot becomes piecewise linear, we can find an empirical estimate for the 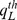 quantile of the subset of the background genes in the IP. This value is chosen depending on the IP because the fraction of background genes (and hence the position of the 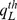 quantile of the subset of background genes in the IP) will depend on the number of background genes (Figure 1).

Normalizing an IP experiment based on an empirical estimate of the 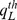 quantile of the background genes to govern the choice of *k* provides a disciplined method of estimating the normalization constant. It suggests that the fraction of true background genes in the subset of size *k* used for normalization will be comparable between IPs hence providing a basis for consistency of the bias of the underestimate of *c_diff_* across IPs and a baseline for comparison of target numbers between IPs. While we find this an appealing property for choosing *k*, we do not claim it has a statistical basis, but this is not critical: the statistical properties of the normalization procedure for any single IP analysis does not depend on the choice of *k*.

## Results and Discussion

### AD Normalization Overcomes a Fundamental Limitation of Other Normalization Methods

For RBPs with large numbers of targets, the model in Equation (EQ2) predicts that median normalization will lead to an underestimate of the number of targets compared to AD normalization. Under median normalization, the null hypothesis for the *i^th^* gene states it has enrichment *μ_i,1_*, in condition 1 and enrichment *μ_i,2_*, in condition 2, the null hypothesis is that
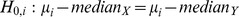
where *median_X_* is the median of the enrichment of genes in condition 1. If condition 1 is an IP, *median_X_* will increase with the number of targets and thus make *H_0,i_* actually valid or at least harder to reject. This places a fundamental limitation on the number of targets an RBP can have when median normalization is used. This limitation is a problem to some degree for all normalization methods that make implicit assumptions about the number of targets an RBP can have.

**Figure 2:**
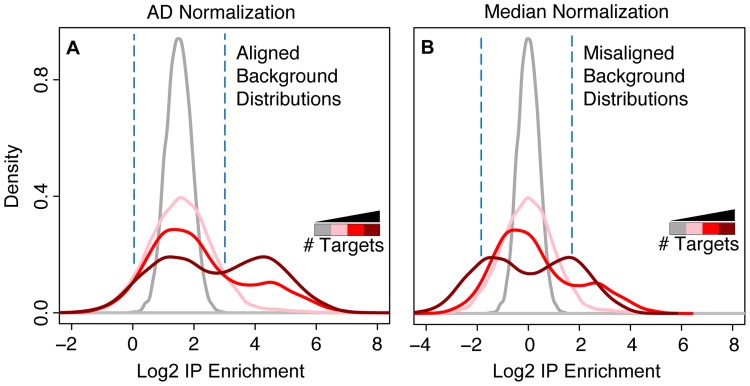
Simulated data illustrates a fundamental advantage of AD normalization. (A) Histograms of simulated IP data for RBPs with an increasing number of targets (pink, red, and dark red lines) and the Mock IP data (gray line), normalized by AD normalization. (B) Same as A, except data was normalized by median normalization. Note how AD normalization properly aligned the portion of each distribution that contained the simulated background data, while median normalization did not, resulting in lower normalized IP enrichment values for the simulated RBP IP targets. The vertical dashed lines have been added to highlight the position of the (Mock) background distribution.

To further explore this theoretical limitation of median normalization relative to AD normalization, we used a simple and idealized model of an IP experiment to simulate data and compared the behavior of AD and median normalization. Even in this idealized scenario, the theoretical limitations of median normalization described above were observed. The simulated data consists of 6,000 total simulated genes. For genes which had no specific IP enrichment (background), we sampled from a normal distribution of mean 0 and for the *bona fide* IP targets, we sampled from a normal distribution of mean 3; both distributions had standard deviation of 1. When AD normalization was applied to the simulated data, it identified and properly aligned the portion of each IP distribution that contained the simulated background data with the Mock (Figure 2). In contrast, median normalization failed to properly align the background distributions with each other or the Mock, resulting in lower normalized IP enrichment values for the simulated RBP IP targets (Figure 2).

Median normalization underestimates the IP enrichment values, especially for simulated RBP IP data containing more simulated targets. Beyond this point, median normalization is unable to distinguish between simulated RBP IP targets and background, resulting in normalized IP enrichment values that clearly fall short of their true enrichment. This places a fundamental limitation on the number of targets an RBP can have when median normalization is used. Since normalization methods like median normalization implicitly make assumptions about the number of targets an RBP has, they will always be subject to this limitation. AD normalization, however, overcomes this fundamental limitation of other normalization methods because it does not assume a preset number of RBP targets.

### The AD Normalization Method Yields Putative Target Sets With a Greater Range of Sizes and Better Concordance with Independent Biological Evidence

In order to compare the AD normalization method with median normalization, both methods were applied to IP data for each of 34 RBPs from a previously published RBP IP data set [4] [GEO:GSE13135, GEO:GSE4393]. After normalization by either the AD method or median centering, the data were analyzed using SAM to identify the putative mRNA targets of the RBPs. The number of targets reported for each RBP by the two methods was correlated (Spearman correlation coefficient 0.83), but the range of sizes of the putative target sets identified by the AD normalization method was substantially greater (Figure S1). This effect was far more pronounced for RBPs with many RNA targets.

**Figure 3:**
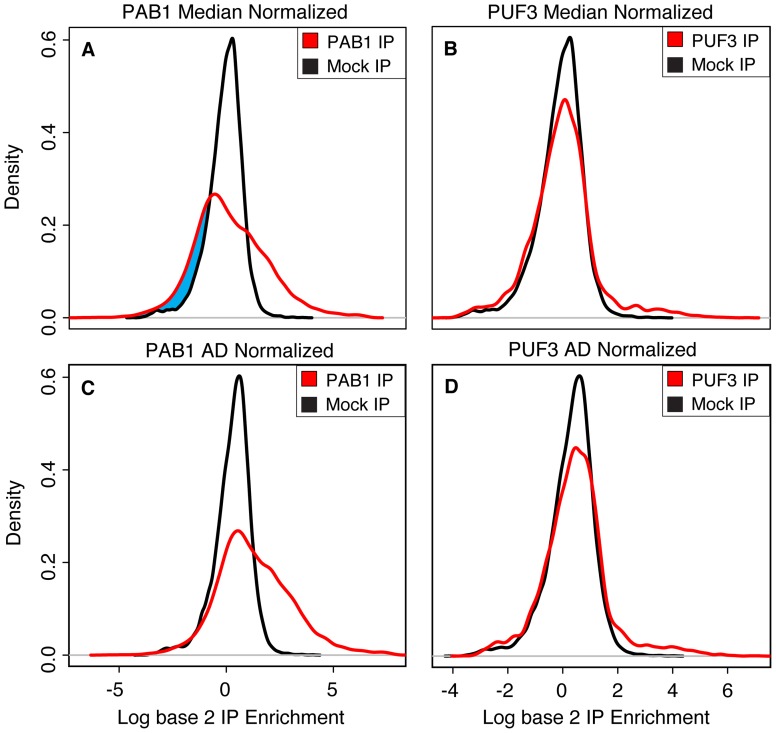
The AD normalization method properly aligns Mock and IP distributions for PAB1 and PUF3. The application of the AD normalization method to IP data from the RBP PAB1 results in greater enrichment relative to the Mock. (A) PAB1 IP data (red line) compared to Mock IP data (black line), both normalized by median normalization. Median normalization results in the dubious situation where there are genes with more negative enrichment values in the PAB1 IP than the Mock IP, as evidenced by the shift of the left-hand side of the PAB1 IP distribution relative to the left-hand side of the Mock IP distribution (highlighted in blue). (B) Same as in part A, except with PUF3 IP data. (C) Same as in part A, except with AD normalized data. Application of AD normalization yields a much more logical outcome where there are no longer more genes with negative enrichment values in the PAB1 IP than the Mock IP. (D) Same as in part C, except with PUF3 IP data, showing that AD normalization does not simply shift every distribution more than median normalization – it properly aligns Mock and IP distributions for RBPs with many targets and with few targets.

The results for the RBP PAB1 highlight this difference. PAB1 is a highly abundant, essential protein that binds to the poly(A) tails of mRNAs; this binding is required for the cap-mediated initiation of translation [16]. Based on the essential role of PAB1 in translation, its high protein abundance, and its broad binding specificity, it has been suggested that PAB1 may bind to nearly all polyadenylated mRNA in the cytoplasm–but previous attempts to identify the mRNA targets of PAB1 using median normalization and SAM were only able to identify ∼1,300 mRNA targets [4] (although the authors provided and made note of strong evidence that PAB1 binds to nearly all mRNAs). Comparison of the PAB1 and mock IP results using median normalization produces an improbable, paradoxical result: more genes are assigned negative enrichment values in the PAB1 IP than the Mock IP (Figure 3). Application of the AD normalization method to the PAB1 IP data, however, results in a more plausible alignment of the Mock and PAB1 IP distributions and greater apparent enrichment in the PAB1 IPs relative to the normalized Mock (Figure 3). Use of the AD normalization method thus enabled the identification of ∼3,500 mRNA targets of PAB1 even at a stringent FDR threshold of 1%, based on SAM (Table 1). This putative target set represents more than 80% of the detectable mRNA transcriptome, which is much more consistent with independent evidence that PAB1 may bind to nearly all polyadenylated mRNA in the cytoplasm [4], [16].

The effect was similar for the RBP PUB1. PUB1 is a highly abundant protein that binds to poly AU regions found in the 3′ untranslated regions of most mRNAs in yeast. Previous work has shown that PUB1 binds to ∼1,000 mRNA targets, and that it is required for the stability of ∼500 mRNAs [4], [12]. Application of the AD normalization method to the same previously published PUB1 IP data resulted in the identification of ∼1,700 targets at an FDR threshold of 1% and ∼3,000 targets at an FDR of 4%, based on SAM. The abundance of PUB1 and the fact that it recognizes a simple sequence motif present in most mRNAs support the results obtained using the AD normalization method and suggest that normalization methods previously used to analyze the PUB1 IP data may have resulted in an underestimate of the number of targets [4].

Despite the fact that AD normalization can result in a significant increase in the number of targets called when applied to RBPs known from other data to have many targets, it does not simply result in more targets called for all RBPs. Specifically, AD and median normalization methods identify similar numbers of targets for RBPs with small, well-defined sets of specific targets, like PUF3 (Table 1). PUF3 is a well-studied RBP that recognizes a specific sequence motif that is highly enriched among its ∼300 targets. The canonical PUF3 recognition sequence motif is present in ∼90% of the known PUF3 targets, and its targets, identified using either the median or AD normalization method, are strikingly enriched for mRNAs that encode proteins localized to the mitochondria. In fact, most of the additional PUF3 targets identified using AD normalization contained the known sequence motif recognized by PUF3, supporting the conclusion that these are *bona fide* PUF3 targets that were missed by the statistical analysis when median normalization was used.

To further assess the differences between AD and median normalization based on independent evidence supporting the targets they each identify, we compared motif enrichment for PUF protein target sets called by SAM (at the same FDR) after either median or AD normalization. We used the Wilcoxon test to calculate p-values for enrichment of mRNAs containing a match to the known sequence motif for each of the PUF proteins (Table S1). For every one of the PUF proteins whose recognition element sequence is known (PUF1-5), the target sets identified using AD normalization followed by SAM were more highly enriched for the known RBP recognition motifs than were the target sets identified from the same data using median normalization. Thus, for RBPs with either narrow or broad target specificity, the AD normalization method yields putative target sets more concordant with independent evidence.

### The AD Normalization Method Is Less Susceptible to False Identification of Targets

In some cases, normalization of RBP IP data with the AD method resulted in fewer RNAs being identified as targets than when median normalization was used (Figure S1). This led us to investigate the number of empirically false targets identified by both methods. To test for false targets, we randomly assigned the data from 6 biological replicate Mock IP arrays into one of two groups (A and B) (the Mock data was previously published here [19] [GEO:GSE22876]). Mock IPs assigned to group A were treated as Mock IP data in this analysis, but Mock IPs assigned to group B were treated as RBP IP data for the purpose of this analysis. The data were normalized using either the median or the AD method, followed by target identification using SAM. Since both group A and group B only contain Mock IP data, any targets identified by either analysis are empirically false targets. When the AD normalization method was used, no false targets were identified, even at a false discovery rate of 20 % (based on SAM) (Table 2). In contrast, when median normalization was used, as many as 51 false targets were identified at a nominal false discovery rate of 0% as scored by the SAM algorithm, and 342 false targets were identified at a 20% FDR (Table 2). These results suggest that the AD normalization method is significantly less susceptible to false positives than median normalization.

**Table 2:** The number of false targets identified from Mock-Mock comparisons after AD or Median normalization.

SAM Reported FDR:		0%	1%	5%	10%	20%
**Median Normalization**						
	Mean	14.8	14.8	18.6	22.7	68.4
	Max	51	51	54	61	342
	Min	0	0	0	0	0
**AD Normalization**						
	Mean	0	0	0	0	0
	Max	0	0	0	0	0
	Min	0	0	0	0	0

The use of median normalization results in the identification of false targets when used in conjunction with SAM, while the AD normalization method does not. The number of false targets resulting from Mock-Mock comparisons using data normalized by either median normalization or the AD method is shown, at various SAM- reported FDRs. No false targets were detected for data normalized by the AD method, at all FDR levels examined. In contrast, as many as 51 false targets were detected at a SAM reported FDR of 1% when median normalization was used. The Mock IP data was previously published in [19].

### The AD Normalization Method is Robust to a Variety of Input Data

**Figure 4:**
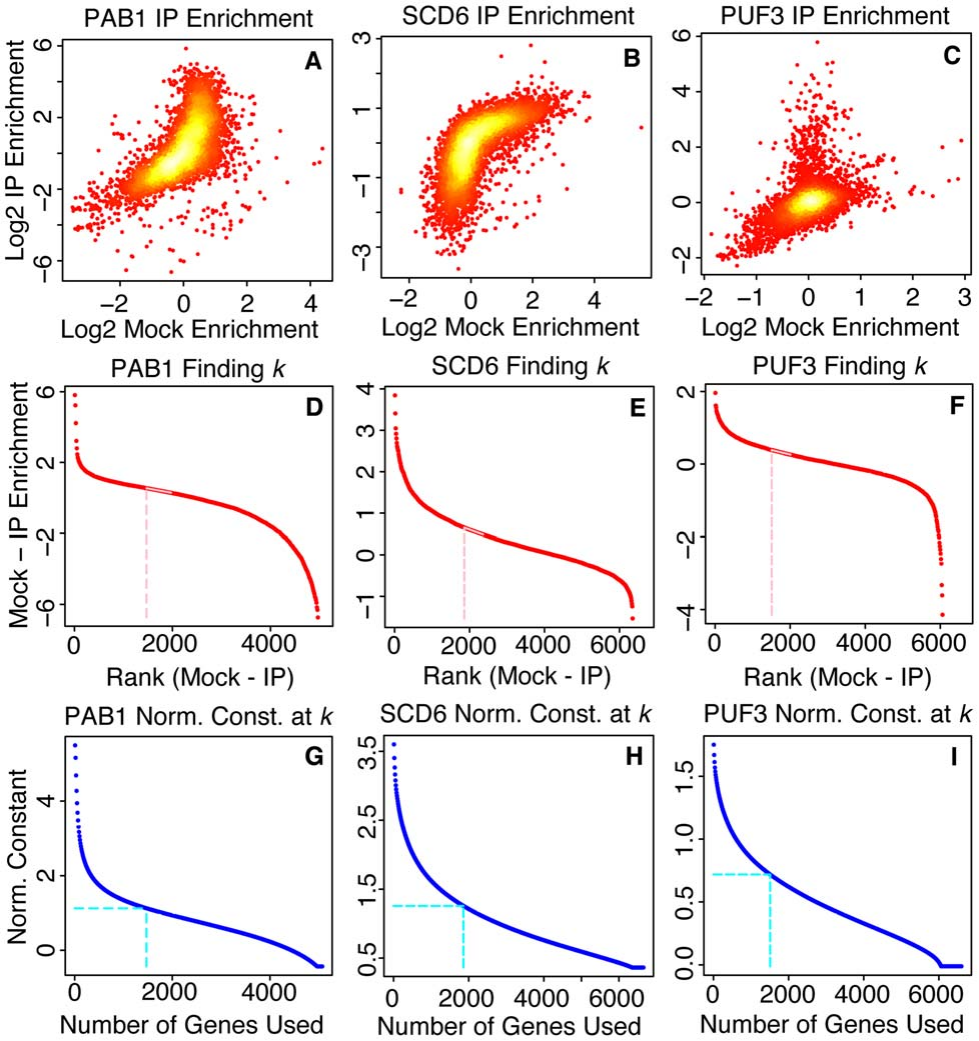
The AD no rmalization method is robust to different types of input data. The method of identifying the normalization constant is robust to different types of input data. (A) A plot of the Log base 2 enrichment values for the RBP IP (y-axis) and the Mock IP (x-axis) for the RBP PAB1. Each point represents a specific gene. (B) Same as A, except for the RBP SCD6. (C) Same as A, except for the RBP PUF3. (D) A plot of each Mock – IP value (y-axis) vs. its rank (x-axis) for the RBP PAB1. Each point represents the Mock value minus the IP value for a specific gene. This plot is used by the AD normalization method to select the number of genes to use for normalization (called *k*). (E) Same as D, except for the RBP SCD6. (F) Same as D, except for the RBP PUF3. (G) A plot of the normalization constant vs. the number of genes in the gene set used for normalization for the RBP PAB1 is shown in blue. The vertical blue dotted line indicates the number of genes to be used for the normalization, chosen by the AD normalization method from the above plot. The horizontal blue dotted line indicates the corresponding normalization constant. This plot is used to find the normalization constant for a given value of *k*. (H) Same as G, except for the RBP SCD6. (I) Same as G, except for the RBP PUF3. The examples shown here are from RBPs with a variety of targets (300–3,000), purified using different reagents, some amplified, some un-amplified, by different experimenters over a span of over 6 years, and using different microarray platforms. Despite these differences, for each sample there was a sufficiently large range of *k* for which 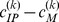 could be modeled linearly.

We evaluated the robustness of our heuristic assumption that there is some sufficiently large range of *k* for which 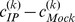 can be modeled linearly. The AD method uses this approximation as part of the algorithm for finding a normalization constant between IP and Mock experiments. We tested this idea by applying the AD normalization method to RBP data from IPs of 3 RBPs, PAB1, SCD6, and PUF3, collected using three different experimental protocols (Figure 4). Each of the three IP data sets was generated by different experimental procedures: the proteins were purified on separate occasions, by different experimenters (more than 6 years apart), using different purification reagents, different types of antibody-coupled beads, different microarray platforms, and different strategies for amplifying and labeling the samples [1], [4], [19] [GEO:GSE13135, GEO:GSE4393, GEO:GSE22876]. After RNA has been purified by an IP, it can either be amplified using T7 RNA polymerase or processed without amplification. Amplification introduces biases but is sometimes necessary. In the tested dataset, the SCD6 IP sample was amplified, while the PAB1 and PUF3 samples were not. In addition, the RBPs PAB1 and PUF3 have been studied in the literature and have widely different numbers of targets: strong experimental evidence indicates that PAB1 has more than 1,000 mRNA targets, while PUF3 has been reported to have ∼300 [1], [4]. The large differences in the input data are evident in Figure 4. Despite these and other differences in the samples and procedures used to generate the data for these analyses, for each sample there was a sufficiently large range of *k* for which 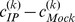 could be modeled linearly. This suggests that the approximations used by the method are robust to considerable variation in the characteristics and sources of error in the data.

## Conclusions

Normalization methods for microarray data have been developed with a focus on the problem of detecting differential gene expression [6], [9], [14], [15]. In such studies, it may be reasonable to use median or quantile normalization in the following cases: when the goal is to identify the genes with the most significant change in relative expression levels, when there is good reason to believe that expression levels of only a small minority of genes are changing, or when the expression levels of a known set of genes (such as ribosomal proteins, ad hoc “housekeeping genes” or other highly expressed genes) is constant across experiments. These and similar assumptions do not apply to microarray data from IP enrichment experiments, where enrichment of each sequence in a specific IP is compared to the enrichment of that sequence in a Mock IP control (modeling the artifacts of the experimental design); doing so necessarily assumes either that the IP has a restricted number of targets or that a particular set of genes are not targets in the IP. In this work, we have provided evidence that median normalization is not, in general, justified for analyzing IP data, necessarily restricts the dynamic range for the number of identifiable targets in the experiment, and is fundamentally limited in its capacity to identify targets.

To our knowledge, no alternative methodology exists for comparative IP enrichment experiments. The essential new feature in our statistical method for determining a normalizing constant is modeling the IP as a linear combination of the background due to the Mock and a non-negative true signal. In this report, we have presented one method for estimating the true signal, but other statistical methods could be used. For example, it would be interesting to investigate the applicability of non-negative factorizations of the data matrix.

The AD normalization methodology yields a substantially larger estimate than median normalization for the number of RNA targets of RNA binding proteins that are independently known to have many targets, such as the poly-A binding protein (PAB1), whose biological characteristics suggest it binds to nearly all polyadenylated mRNA in the cytoplasm. For RBPs that bind to a small number of RNAs, such as PUFs 1–5, using the AD normalization method identifies target sets that are comparable in number to those identified by median normalization, but with better enrichment of the known recognition sequence motifs in the target sets, suggesting more accurate performance. In addition, based on a control analysis of Mock data, AD normalization results in far fewer false positives than were obtained with median normalization. Overall, the adaptive (AD) normalization method is less biased and results in better concordance with biological evidence.

The AD normalization methodology we describe here, although illustrated using RNA-IP data, may have much broader applications – its general utility is to data analysis challenges where the goal is to detect and identify a specific subset of a large set of discrete species, enriched or depleted, compared to a corresponding negative control procedure, when the sample is subjected to a selective treatment or fractionation procedure. Although the method is not specific to immunoprecipitation as a fractionation procedure or nucleic acids as the species fractionated, application to IP experiments using next generation sequencing in place of microarrays to identify differentially enriched sequences is one obvious extension. Adapting our normalization method so that it can be applied to sequencing data (such as from a CLIP-Seq experiment) is straightforward. First, divide the genome or transcriptome into bins (using the same bins for the Mock and the IP data). Next, count the number of reads falling into each bin, and retain those bins with counts in both the IP and Mock. If desired, sequencing bias can be accounted for by subjecting a total RNA reference sample to the same binning procedure, and the IP and Mock data can be divided by the reference counts. Finally, log transform these data and input them into the method described in our paper. The resulting normalization constant can then be used to normalize all of the read counts in the IP data. It is not necessary to have a sample of total RNA as a reference, so the adaptation to single color microarray data (such as the Affymetrix platform) is also possible after it is log transformed. Regardless of the platform, an accurate measure of the background (i.e. a Mock experiment) is required.

Our normalization method can be applied to any log transformed data analogous to an IP and a Mock. In addition, the modeling in this paper is performed for data assumed to be normalized by an additive constant. The conceptual framework can be applied more generally, such as to developing models for data where multiplicative normalization is required. Further, while our specific focus in this work was motivated by our interest in characterizing RNA-protein interactions, its potential applications are wider; indeed, we believe that its potential applications are not restricted to genomic or even biological data.

## Supporting Information

Figure S1 **Number of targets called by SAM after median or AD normalization for 34 different RBPs.** The AD normalization method yields IP enrichment values and putative target sets with greater range than median normalization. This file contains a plot of the number of targets that are called by SAM after median normalization on the y-axis vs. the number of targets that are called by SAM after AD normalization on the x-axis for each of 34 RBPs from a previously published RBP IP dataset [4]. Each point represents an RBP. The dashed blue line is the trend line for the data, which has a Spearman correlation coefficient of 0.83. The dashed red line is the line y  =  x. When the data is normalized by the AD normalization method, there is a much greater range in the number of targets called by SAM (at a SAM reported FDR of 1%). This effect is especially pronounced for RBPs with many targets.
(PDF)

Table S1 **Enrichment of known Puf sequence motifs for target sets resulting from AD or median normalization.** This file contains a table of PUF Motif Enrichment For PUF Target Sets Resulting From AD or Median Normalization. Shown in the table are the Wilcoxon p-values for enrichment of mRNAs that contain the recognition motif for the given RBP in target sets defined by SAM (at the same FDR) after either median or AD normalization. The use of AD normalization followed by SAM results in RBP target sets with greater enrichment of known RBP recognition motifs for the PUFs (PUF1-5), compared to median normalization.
(PDF)

File S1 **An R script for performing AD normalization.** This file contains an R script encoding a function for performing AD normalization. This file can be opened with a simple text editor and it can be run in R, the language and environment for statistical computing and graphics.
(R) Click here for additional data file.
