# Supplementary figures and images for: Improved Discovery of Molecular Interactions in Genome-Scale Data with Adaptive Model-Based Normalization
Source: PLoS One. 2013 Jan 22;8(1):e53930. doi: 10.1371/journal.pone.0053930 (PMC3551948; doi:10.1371/journal.pone.0053930)

Number of Targets Called By SAM (1% FDR)

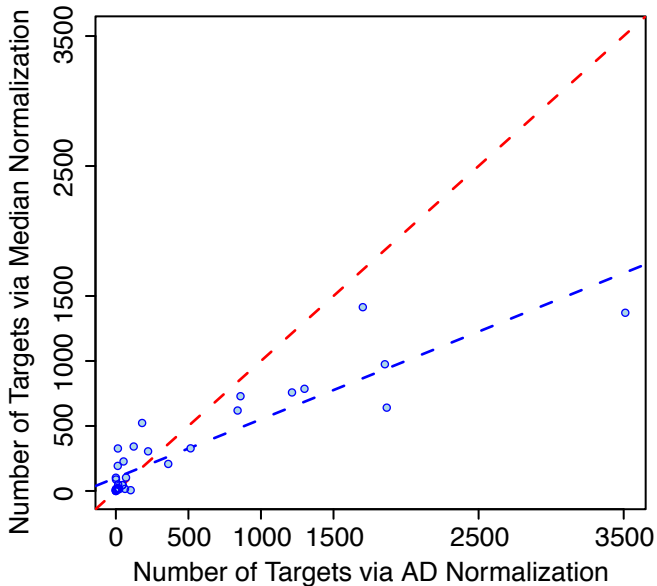

Supplement: Figure S1 — Number of targets called by SAM after median or AD normalization for 34 different RBPs. The AD normalization method yields IP enrichment values and putative target sets with greater range than median normalization. This file contains a plot of the number of targets that are called by SAM after median normalization on the y-axis vs. the number of targets that are called by SAM after AD normalization on the x-axis for each of 34 RBPs from a previously published RBP IP dataset [4]. Each point represents an RBP. The dashed blue line is the trend line for the data, which has a Spearman correlation coefficient of 0.83. The dashed red line is the line y = x. When the data is normalized by the AD normalization method, there is a much greater range in the number of targets called by SAM (at a SAM reported FDR of 1%). This effect is especially pronounced for RBPs with many targets. (PDF) [file pone.0053930.s001.pdf]
