# Supplementary material for: Improved Discovery of Molecular Interactions in Genome-Scale Data with Adaptive Model-Based Normalization
Source: PLoS One. 2013 Jan 22;8(1):e53930. doi: 10.1371/journal.pone.0053930 (PMC3551948; doi:10.1371/journal.pone.0053930)
Supplement: Table S1 — Enrichment of known Puf sequence motifs for target sets resulting from AD or median normalization. This file contains a table of PUF Motif Enrichment For PUF Target Sets Resulting From AD or Median Normalization. Shown in the table are the Wilcoxon p-values for enrichment of mRNAs that contain the recognition motif for the given RBP in target sets defined by SAM (at the same FDR) after either median or AD normalization. The use of AD normalization followed by SAM results in RBP target sets with greater enrichment of known RBP recognition motifs for the PUFs (PUF1-5), compared to median normalization. (PDF) [file pone.0053930.s002.pdf]

	Enrichment of Motif	
	Median Norm	AD Norm
PUF1	3e-2	1e-2
PUF2	1e-12	7e-22
PUF3	2e-112	3e-119
PUF4	5e-90	6e-93
PUF5	3e-37	1e-39
